# Optimizing immunomagnetic separation for efficient *E. coli* O157:H7 recovery and detection

**DOI:** 10.1186/s13568-025-01991-6

**Published:** 2025-12-20

**Authors:** Assem Abolmaaty, Abrar AbuHamdia, Dina H. Amin

**Affiliations:** 1 Department of Food Science, Faculty of Agriculture, Ain Shams University, Cairo, Egypt; 2 Division of Genomics and Precision Medicine, College of Health and Life Sciences, Hamad Bin Khalifa University, 34110 Doha, Qatar; 3 Department of Microbiology, Faculty of Science, Ain Shams University, Cairo, Abbasyia Egypt

**Keywords:** *E. coli* O157:H7, Immunomagnetic separation, Ground beef, IgG concentration, Antibody-coated beads, Bacterial detection

## Abstract

Immunomagnetic separation is an invaluable tool in biomedical and food safety applications due to their high specificity and efficiency in capturing target cells. This study is a comprehensive investigation of the Ag-Ab reaction and the effects of several factors such as IgG concentration, incubation temperature, and incubation time on the binding efficiency of target cells to the immunomagnetic beads aiming to maximize captured *E. coli* O157 cells from ground beef samples. Our findings reveal a concentration-dependent relationship between IgG dilution and binding efficiency, with lower dilutions (1:100) yielding higher binding percentages. Additionally, the optimal incubation temperature for bead-cell interactions was identified as 32 °C, with deviations above or below this temperature impairing binding efficiency. Longer incubation times (up to 120 min) significantly improved antibody coating on the beads, enhancing their ability to capture target cells. The study also demonstrated a strong correlation between bacterial load and binding efficiency, highlighting the sensitivity of the immunomagnetic separation method in detecting *E. coli* O157 even at low concentrations. These findings underscore the importance of precisely controlling experimental parameters to maximize the efficiency and specificity of immunomagnetic separation techniques, providing a valuable framework for researchers aiming to refine bead-based cell capture protocols for diagnostic or research applications.

**Supplementary Information:**

The online version contains supplementary material available at 10.1186/s13568-025-01991-6.

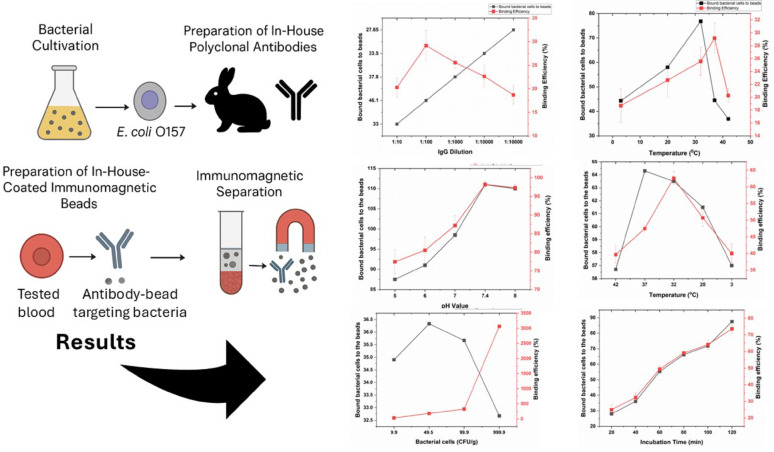

## Introduction

Shiga toxin-producing *Escherichia coli* (STEC), also referred to as verocytotoxin-producing *E. coli*, are a group of pathogenic bacteria responsible for severe foodborne illnesses, including hemorrhagic colitis (HC) and hemolytic uremic syndrome (HUS). Among the STEC serogroups, O157, O26, O103, O111, and O145 are commonly associated with outbreaks, with *E. coli* O157 being the most prevalent (Paton and Paton 2004). This particular serogroup has been linked to numerous foodborne outbreaks, including two major cases in Michigan and Oregon caused by undercooked hamburgers. Since then, over 100 outbreaks have been documented globally, emphasizing the public health significance of this pathogen. *E. coli* O157 stands out due to several virulence factors, most notably Shiga toxins 1 and 2 (stx1 & stx2), which play a central role in the development of HC and HUS in humans (Nada et al. 2023).

The primary reservoir for *E. coli* O157 is cattle, and the pathogen is commonly found in foods of bovine origin as well as other food and water sources contaminated with bovine feces. Contaminated food products, including ground beef, roast beef, raw milk, cider, yogurt, cheese, fermented sausage, cooked maize, lettuce, and sprouts, have been identified as vehicles for transmission (Rangel et al. 2005; Lim et al. 2010). Notably, undercooked ground beef has been implicated in most of the *E. coli* O157 outbreaks, with an infective dose estimated to be as low as 100 cells (Pennington 2010). The combination of a low infective dose, improper cooking, and high consumption of ground beef has contributed significantly to the pathogen’s frequent outbreaks. Therefore, rapid and sensitive detection methods for *E. coli* O157 in raw ground beef are critical for food safety (Wang et al. 2021).

Traditional detection methods rely on selective growth media, such as Sorbitol MacConkey (SMAC) agar and TC-SMAC (SMAC with Cefixime and Potassium Tellurite), followed by biochemical and serological tests to confirm the presence of *E. coli* O157 (March and Ratnam 1986). While effective, these methods are time-consuming, often taking 5–6 days to deliver conclusive results. In response to these limitations, advanced detection techniques have been developed, including DNA probe methods, latex agglutination assays, enzyme-linked immunosorbent assays (ELISA), immunoprecipitation, and polymerase chain reaction (PCR) (Feng 1995; Bettelheim 2003). These methods offer enhanced sensitivity and specificity, yet their efficiency can still be improved. Several studies have focused on optimizing specific conditions such as temperature, pH, reagent concentrations, and incubation periods—to improve the performance of these techniques (Paton and Paton 1998). However, there remains a gap in the literature as no comprehensive study has provided a fully optimized protocol that accounts for all key variables across the detection process.

Among the advanced methods, PCR has emerged as a powerful tool for screening specific microorganisms by detecting unique genes associated with the target pathogen (Paton and Paton 1998). PCR is often preceded by an enrichment step to promote the growth of the target microorganism, thereby increasing the assay’s sensitivity. In some cases, PCR can detect as little as 1 colony forming unit (CFU) of a microorganism per gram of food (Feng 1995). However, the detection of *E. coli* O157 in complex food matrices, such as ground beef, presents significant challenges due to the presence of background microflora and PCR inhibitors. Ground beef often contains high levels of non-target bacteria, reaching up to 10⁶ CFU/g, while the levels of *E. coli* O157 can be extremely low. Additionally, PCR inhibitors like proteins, lipids, salts, and heme may interfere with the DNA polymerase activity, reducing the amplification efficiency and leading to false-negative results (Wilson 1997). Therefore, the efficient separation of *E. coli* from food samples and the removal of interfering substances are critical for accurate detection.

One promising technique for improving the isolation of *E. coli* O157 from complex food samples is immunomagnetic separation (IMS). This method involves using magnetic beads coated with antibodies that specifically bind to antigens on the target bacteria (Chapman et al. 1994). Once bound, the beads and the attached bacteria can be separated from the sample using a magnetic field, effectively removing unwanted microflora and PCR inhibitors. The success of IMS depends on several test conditions, including the incubation period, pH, and temperature. Most studies have followed general conditions of 12–25 h of incubation, a pH of 8, and temperatures of 33–37 °C (Feng 1995). However, the optimal conditions for maximizing the binding and separation efficiency have not been thoroughly explored or standardized across different studies.

The introduction and advancements in immunomagnetic separation (IMS) have enabled the rapid, sensitive, precise, and reliable detection of various pathogens in food matrices. Enhanced magnetic nanobeads functionalized with specific antibodies or aptamers have been used to detect *E. coli* O157:H7, *Listeria monocytogenes*, and *Salmonella* spp. in complex food matrices with high sensitivity and selectivity (Wang et al. 2018; Chen et al. 2021). Similarly, nanostructured IMS platforms have demonstrated rapid pathogen recovery and detection performance comparable to PCR-based assays (Zhang et al. 2017; Wu et al. 2014). More recently, multifunctional magnetic nanoparticles incorporating optimized antibody orientation and surface blocking strategies have shown improved analytical performance for bacterial capture and quantification (Liu et al. 2023). The efficiency and increased speed of pathogen detection provided by IMS contribute to the rapid identification of foodborne pathogens, even at very low concentrations, enabling timely interventions, preventing the spread of foodborne diseases, and protecting public health. Nonetheless, further optimization studies are needed to standardize parameters affecting binding efficiency and analytical sensitivity, and to enhance diagnostic techniques.

The primary objective of this study is to address these knowledge gaps by developing an optimized protocol for producing in-house polyclonal antibodies and integrating them into magnetic beads for the efficient isolation and detection of *E. coli* O157 in ground beef. This research will systematically evaluate the impact of critical test conditions such as temperature, pH, incubation period, and the concentrations of both antibody and antigen on the binding efficiency of *E. coli* to the coated beads. After determining the optimal conditions, the study will apply them to isolate *E. coli* from ground beef samples. The ultimate goal is to maximize capturing protocol for an efficient detection of *E. coli* O157 in complex samples such as ground beef, contributing to improved food safety and public health protection. Our study focuses on in-house polyclonal IgG optimization in ground beef, rather than using pre-optimized commercial kits. This approach demonstrates cost-effective alternatives for local laboratories in developing countries.

## Methodology

### Bacterial cultivation

*Escherichia coli* O157 strain C9490 (obtained from the Centers for Disease Control, Atlanta, GA, USA) was cultivated in Tryptic Soy Broth (TSB) supplemented with 0.5% dextrose. Cultures were grown overnight at 37 °C with rotary agitation (200 rpm) in 250 mL baffled flasks containing 100 mL of medium. Cells were harvested by centrifugation at 10,000 xg for 10 min at 4 °C (Ahmed et al. 2020; Hyeon et al. 2018). The harvested cells were resuspended in sterile phosphate-buffered saline (PBS) with bovine serum albumin (BSA), pH 7.4, and diluted to the required cell densities. Cell density was measured using both spectrophotometric methods and a Petroff-Hausser bacterial counting chamber.

### Preparation of in-house polyclonal antibodies from rabbit serum

#### Initial treatment of rabbits

New Zealand white Rabbits were obtained from Millbrook Farms, Amherst, MA. The rabbits were held for several days in the facility to acclimate them. An initial bleeding was done on new rabbits to test pre-immune serum. New rabbits were also tranquilized with an intramuscular injection of 0.25 ml ketamine HCl (100 mg/ml) and 0.25 ml Xylazine (20 mg/ ml) for 6–8 Ib. rabbit, (0.5 ml Ketamin & 0.5 ml Xylazine for 8–12 Ib. rabbit) (Sayedahmed 2006).

#### Preparation of E. coli O157 antigen and immunization of rabbits

A 1 L overnight culture of *E. coli* O157 was prepared and harvested as previously described. The harvested cells were inactivated by treatment with 4% formaldehyde at 20 °C overnight. Following inactivation, the cells were washed three times with 30 mL of phosphate-buffered physiological saline (0.01 M K₂HPO₄, 0.01 M NaH₂PO₄, and 0.85% NaCl, pH 7.4) to remove any residual formaldehyde.

New Zealand White female rabbits were immunized by intradermal injection along the dorsal surface. Each rabbit received 10 injections of 0.05 mL of the *E. coli* cell suspension, containing 4 × 10⁹ and 4 × 10^1^⁰ cells/mL in sterile 0.85% NaCl. The suspension was supplemented with 0.05% aluminum hydroxide (Al(OH)₃) as an adjuvant, which was prepared following the protocol outlined by Harlow and Lane (1988) and sterilized via autoclaving. The immunization and booster injection protocols were also conducted in accordance with Harlow and Lane (1988).

#### Isolation of anti-E. coli antibodies from rabbits serum

Ten days following the initial injection with cell suspensions, blood was collected from the rabbits (Sayedahmed 2006). The animals were placed in restraining cages, and the ear was shaved and disinfected with 70% alcohol. Blood was drawn using a 20-gauge needle attached to tygon tubing, with one end inserted into the ear's blood vessel and the other connected to a vacutainer serum separator tube (Morrill Antibody Production Center, Animal Care Department, University of Massachusetts at Amherst). A total of 52 mL of blood was drawn, followed by an additional 2–3 mL, ensuring that the total volume did not exceed 8 mL per kilogram of the rabbit’s body weight. After the procedure, the needle was removed, and pressure was applied to the blood vessel to stop the bleeding. The ear was wiped again with alcohol, and mineral oil was applied to the skin to promote healing. Subsequent booster injections were administered biweekly following the initial boost, in accordance with Harlow and Lane (1988), to monitor the development of anti-*E. coli* O157 antibodies in the rabbit serum.

#### Purification of anti-* Escherichia coli*O157:H7 polyclonal antibodies

Protein A agarose column (2.5 mL packed volume, Sigma, cat. no. P-2545) was equilibrated with binding buffer (0.02 M NaH₂PO₄, 0.15 M NaCl, and 0.025 M NaN₃) using gravity flow. The rabbit anti-*E. coli* O157 antiserum, as described previously, was collected, and 1.0 mL of the antiserum was passed through the column three times to ensure efficient binding. The column was then washed with 10 volumes of binding buffer to remove unbound proteins.

IgG was eluted using five column volumes of elution buffer (0.2 M Na₂HPO₄ and 0.1 M citric acid, pH 3.0), and 2.0 mL fractions were collected. Fractions showing absorbance at 280 nm (totaling 11 tubes) were pooled and neutralized with 0.1 N NaOH. The purified IgG was quantified using a spectrophotometric agglutination assay, and the protein concentration was determined by the Lowry method (1951), with bovine serum albumin (Sigma) serving as the standard. After elution, the column was regenerated by re-equilibrating with 25 volumes of binding buffer.

### Preparation of in-house-coated-immunomagnetic beads

#### Preparation of the reagents

Polystyrene microsphere beads, with diameters ranging from 0.6 to 0.9 μm and at a concentration of 5% w/v, were obtained from Bang Laboratories, Inc. (Carmel, IN). The beads were used as received, without additional cleaning. The coat/wash buffer was prepared by combining 470 mL of Solution A [27.6 g of NaH₂PO₄·H₂O in 1 L of distilled water] with 30 mL of Solution B [28.4 g of anhydrous Na₂HPO₄ in 1 L of deionized water]. The pH was adjusted to 5.5 using either 0.5 M HCl or 0.5 M NaOH, and the final volume was brought to 1 L with deionized water (Sayedahmed 2006).

#### Procedure for IgG coating beads

Polystyrene microsphere beads with a diameter of 0.6–0.9 μm and a concentration of 5% w/v were purchased from Bang Laboratories, Inc. (979 Keystone Way, Carmel, IN 46032-2823). Polystyrene microsphere beads were agitated thoroughly before use to ensure uniform suspension. The antibody solution was prepared by dissolving 200 μg of IgG in 4 mL of coat/wash buffer at pH 7.8. The wash/storage buffer consisted of 0.01 M NaH₂PO₄·H₂O in 0.9% NaCl at pH 7.8. A 0.2 mL aliquot of the microsphere suspension was mixed with 2.0 mL of antibody solution in a microcentrifuge tube. The mixture was incubated at room temperature for 1 h with periodic mixing, then centrifuged at 10,000 g for 30 min. After discarding the supernatant, the pellet was resuspended in 2.0 mL of coat/wash buffer and centrifuged again under the same conditions. The supernatant was discarded, and the pellet was resuspended in 0.5 mL of wash/storage buffer, transferred to a fresh microcentrifuge tube, and centrifuged at 11,000 rpm for 3 min. The supernatant was removed, and the pellet was resuspended in 2.0 mL of wash/storage buffer, with 0.1% sodium azide added for long-term storage if necessary.

#### Coating of immuno-magnetic beads with E.coli O157:H7

Immuno-magnetic beads coated with goat anti-rabbit IgG were obtained from BioMag Laboratories. Each milligram of BioMag suspension, at a concentration of 1 mg/mL, contains 5 × 10⁸ magnetic particles. As BioMag products are not supplied sterile and contain azide, the magnetic particles were washed three times in sterile glass vials with plastic caps using PBS buffer containing 5% BSA. To prepare for cell sorting, 0.1 mL of the immuno-beads were suspended in a total volume of 2.0 mL of the PBS/BSA buffer and mixed using a Vortex-2 Gene shaker. The beads were then concentrated magnetically to one side of the vial and washed three additional times with the same buffer (Sayedahmed 2006).

To bind the immuno-magnetic beads to rabbit anti-*E. coli* O157, 50 μL of rabbit IgG (containing 182.149 μg of specific IgG) was added to 2.0 mL of PBS/BSA buffer, and the mixture was combined with the washed beads. The vials were placed on a motorized rotating plate (Multi-Purpose Rotator, Model 150 V, Scientific Industries Inc.) connected to a timing unit (Lab Controller, Fisher Scientific Co.). The vials were rotated for 1 min at 7 rpm once every 10 min. After binding, the beads were magnetically concentrated to one side of the vial and washed three times with the same buffer.

#### Optimized IgG coating and immunomagnetic separation (IMS) protocol

To ensure reproducibility and highlight the practical applicability of the optimized immunomagnetic separation (IMS) process, a detailed protocol for the coating of magnetic beads with rabbit polyclonal IgG and subsequent capture of *E. coli* O157:H7 was developed (Sayedahmed 2006) as follows.

#### Activation and washing of magnetic beads

Commercially available beads were washed three times with phosphate-buffered saline (PBS, 0.01 M, pH 7.4) containing 0.05% (v/v) Tween-20 to remove preservatives and equilibrate the surface. Magnetic separation was used between each washing step to ensure complete removal of supernatant.

#### Antibody coupling

Purified rabbit polyclonal IgG raised against *E. coli* O157:H7 was prepared at the optimized concentration of 20 µg/mL in PBS (pH 7.4). The washed beads were incubated with the IgG solution at room temperature (25 °C) for 30 min under gentle rotation (15 rpm) to allow optimal antibody binding via the Protein A sites. After incubation, unbound antibodies were removed by magnetic separation followed by three washes with PBS.

#### Blocking of non-specific sites

To minimize non-specific adsorption during bacterial capture, the IgG-coated beads were incubated with 5% (w/v) bovine serum albumin (BSA) prepared in PBS at pH 8.0 for 1 h at 25 °C with mild agitation. This step effectively blocked residual binding sites on the bead surface and stabilized the antibody layer. The beads were subsequently washed twice with PBS to remove excess BSA.

#### Storage of IgG-coated beads

The coated beads were suspended in PBS containing 0.1% BSA and stored at 4 °C until use. Under these storage conditions, the beads maintained their binding efficiency for up to two weeks without detectable loss of antibody activity.

#### Immunomagnetic separation (IMS) of E. coli O157:H7

Ground beef samples (25 g) were homogenized with 225 mL of sterile PBS (0.01 M, pH 7.4). Aliquots (1 mL) of the homogenate were inoculated with known concentrations of *E. coli* O157:H7 (ranging from 10^1^ to 10⁶ CFU/mL). Subsequently, 1 mg of IgG-coated beads was added to each 1 mL sample and incubated for 20 min at 32 °C with gentle rotation (10 rpm) to facilitate antigen–antibody binding.

Following incubation, the beads were magnetically separated for 2 min and washed three times with PBS to eliminate unbound bacteria. Captured bacterial cells were resuspended in 100 µL PBS and plated on selective agar for viable cell enumeration.

#### Quantification of bound bacteria

Quantification of *E. coli* O157:H7 captured by the IgG-coated magnetic beads was performed using plate enumeration. After the immunomagnetic separation (IMS) step, bead–bacterium complexes were magnetically separated and resuspended in 1.0 mL of sterile phosphate-buffered saline (PBS). Serial dilutions were prepared, and 0.1 mL aliquots from each dilution were spread onto Sorbitol MacConkey agar supplemented with cefixime and tellurite (CT-SMAC) and incubated at 37 °C for 24 h. The resulting colonies were counted, and the data were expressed as colony-forming units (CFU) per milligram of magnetic beads.

Each measurement was performed in triplicate, and mean values were used to calculate binding efficiency at different inoculum concentrations. The relationship between bacterial concentration and the number of bound bacteria was used to evaluate the binding performance of the optimized IMS system.

#### Measuring the bounded IgG to the beads and the bounded cells

To measure the binding of IgG to the beads and the adherence of cells, a final cell density of 1 × 10^3^ cells/mL (unless otherwise specified) was introduced to the IgG-coated beads in a total volume of 2.0 mL of PBS/BSA buffer. The reaction mixture was incubated under various temperature and time conditions, with intermittent agitation as described earlier. Following incubation, the beads were magnetically concentrated to one side of the vial.

The beads were resuspended in 1.0 mL of PBS, and 0.1 mL of the suspension was plated onto Violet Red Bile Agar (VRBA) plates, which were then incubated overnight. Colony counts of *E. coli* O157 on these plates were used to estimate the total amount of IgG bound to the beads or the number of cells attached to the coated beads, allowing for optimization of the binding process. Control experiments were conducted to assess the impact of the glass vials, incubation conditions, and the presence of IgG on the plate counts (Sayedahmed 2006).

#### Optimization of the assay

To maximize the antibody-antigen binding capacity, the assay was optimized by systematically varying key parameters: temperature, IgG concentration, incubation time, pH, and cell density. Each variable was adjusted individually while keeping the others constant. Once the optimal conditions for a specific variable were identified, they were applied in subsequent experiments to refine the assay further (Liu et al. 2014).

### Detection of E. coli O157:H7 in ground beef using in-house-coated-immunomagnetic beads

#### Preparation of ground beef samples and testing for the absence of E. coli O157:H7

Ground beef with 5% fat content was procured from a local retail store. The purchased samples were tested for the presence of *E. coli* and other verotoxins, including SLT-1 and SLT-2, using biochemical, immunological, and genotyping methods. Ten grams of ground beef were mixed with 90 ml of Tryptic Soy Broth containing 0.5% dextrose in a Whirl–Pak stomacher bag with an inserted mesh (NASCO, Fort Atkinson, WI, USA). The mixture was processed in a Stomacher 400 BA 7021 (Seward, Tekmar, Cincinnati, OH, USA) at 230 rpm for 90 s. The homogenate was transferred to a 1-L flask and incubated with rotary agitation at 300 rpm and 37 °C for 24 h. After incubation, the culture was centrifuged at 1000 rpm for 3 min to remove large debris. A loop of the supernatant was streaked onto Violet Red Bile Agar (VRBA) plates and incubated overnight at 37 °C. Typical purple colonies were isolated and transferred to Tryptic Soy Agar (TSA) slants for further incubation at 37 °C overnight. The growth from these slants was then tested using latex polystyrene beads, a spectrophotometric immuno-agglutination assay, an ELISA assay (Ashkenazi and Cleary 1989) for *E. coli* O157 serotype detection, and PCR for the corresponding genes.

#### Inactivation of E. coli using freezing and thawing process

Ground beef that tested negative for *E. coli* O157:H7 and other verotoxin-producing SLT-1 and SLT-2 was aseptically divided into plastic ziplock bags in a thin layer (0.5 cm). The bags were then frozen and subjected to at least three freeze–thaw cycles using liquid nitrogen before being stored at −20 °C for future use. Aseptically, one sample was thawed, and 10 g was added to 90 ml of sterile Tryptic Soy Broth (TSB +) in a stomacher bag with an inserted mesh. The stomacher bags were processed at 230 rpm for 90 s. A loop of the filtrate was streaked onto Violet Red Bile Agar (VRBA) plates, which were incubated at 37 °C for 24 h. Successful freeze–thaw processing was indicated by the absence of colonies on the VRBA plates. Additionally, 10 ml of the filtrate was transferred to 100 ml of TSB + in 250 ml baffled flasks and incubated for 24 h at 37 °C. The growth was subsequently tested for *E. coli* O157:H7 using latex polystyrene beads, a spectrophotometric immuno-agglutination assay, an ELISA assay for SLT-1 and SLT-2, and PCR for VT1 and VT2 genes (Thayer & Boyd 1999).

#### Seeding E. coli into the ground beef samples

Cell suspensions with varying cell concentrations (10, 50, 100, 200, and 500 cells, prepared in 0.2 ml PBS) were introduced into 10 g samples of ground beef. The bacterial cells were allowed to bind for 15 min at 5–8 °C. Subsequently, homogenates and filtrates were prepared using differential centrifugation and coffee filter method as described previously. The resulting pellets were resuspended in 30 ml of Tryptic Soy Broth (TSB) supplemented with 0.5% dextrose, and the suspensions were transferred to 250 ml baffled flasks. These flasks were incubated with rotary agitation (300 rpm) at 37 °C for various durations (3.5, 4.5, and 5.5 h). Following incubation, the cultures were centrifuged at 16,000 g for 10 min at 4 °C to harvest the cells. The pellets were then resuspended in 1.0 ml of distilled water and 0.1 ml of the suspension was plated onto Violet Red Bile Agar (VRBA) plates (Sayedahmed 2006).

#### Extraction of E. coli O157:H7 from ground beef using different extraction solutions

Ground beef samples (10 g) were placed in stomacher bags with 90 ml of various extraction solutions, including a stomaching buffer at pH 7.0, 0.01 M KCl with Tween-20, a 0.1 M buffer at pH 6.0, a 0.01 M buffer at pH 7.4, and PCR buffer. The samples were processed in a stomacher at low speed (170 rpm) for 60 s at room temperature, and the resulting filtrates were passed through paper coffee filters using a vacuum pump. The transparency of the filtrates was measured at 600 nm, and the extraction solution producing the clearest supernatant was selected for further use. In the optimization phase for detecting *Escherichia coli* O157, homogenates of unseeded frozen ground beef, both with and without enrichment media, were plated on Violet Red Bile Agar (VRBA) plates and incubated at 37 °C for 24 h, with no colonies of *E. coli* detected. Filtrates were enriched in Tryptic Soy Broth (TSB +) at 37 °C for 24 h and subsequently tested using latex agglutination, spectrophotometric immuno-agglutination, ELISA, and PCR assays, all of which confirmed the absence of *E. coli* O157, indicating that the freeze–thaw process was successful, and the ground beef samples were free from both viable and non-viable bacteria (Sayedahmed 2006).

### Statistical analysis

A full Design of Experiments (DoE) approach was not applied in this study. Instead, a one-variable-at-a-time (OVAT) method was employed to independently evaluate the effects of each parameter (IgG concentration, incubation time, temperature, and pH) on binding efficiency. This approach was chosen due to limited resources and the exploratory nature of this preliminary optimization. Future studies will incorporate a factorial DoE strategy to assess potential interactions among variables and enhance model robustness.

All tables and figures have been reformatted to include descriptive statistical information. Data are expressed as mean ± standard deviation (SD) from three independent replicates (n = 3).

The binding efficiency (%) was calculated according to the following equation:

$$\mathrm{\%}\text{ Binding}=\frac{\text{Captured cells}}{\text{Total cells added}}\times 100$$

## Results

### Optimization of in-house rabbit anti-E.coli-coated immunomagnetic beads for detecting* Escherichia coli* O157:H7 in ground beef

#### Effect of IgG concentration on the binding of cells to beads

At zero time, the mean CFU counts were 140 with glass vials and 148 with naked beads (without IgG). Values were adjusted by subtracting baseline counts from experimental counts. Control experiments confirmed no effect of glass vials, beads, temperature, or IgG on CFU counts*.*

**Fig. 1:**
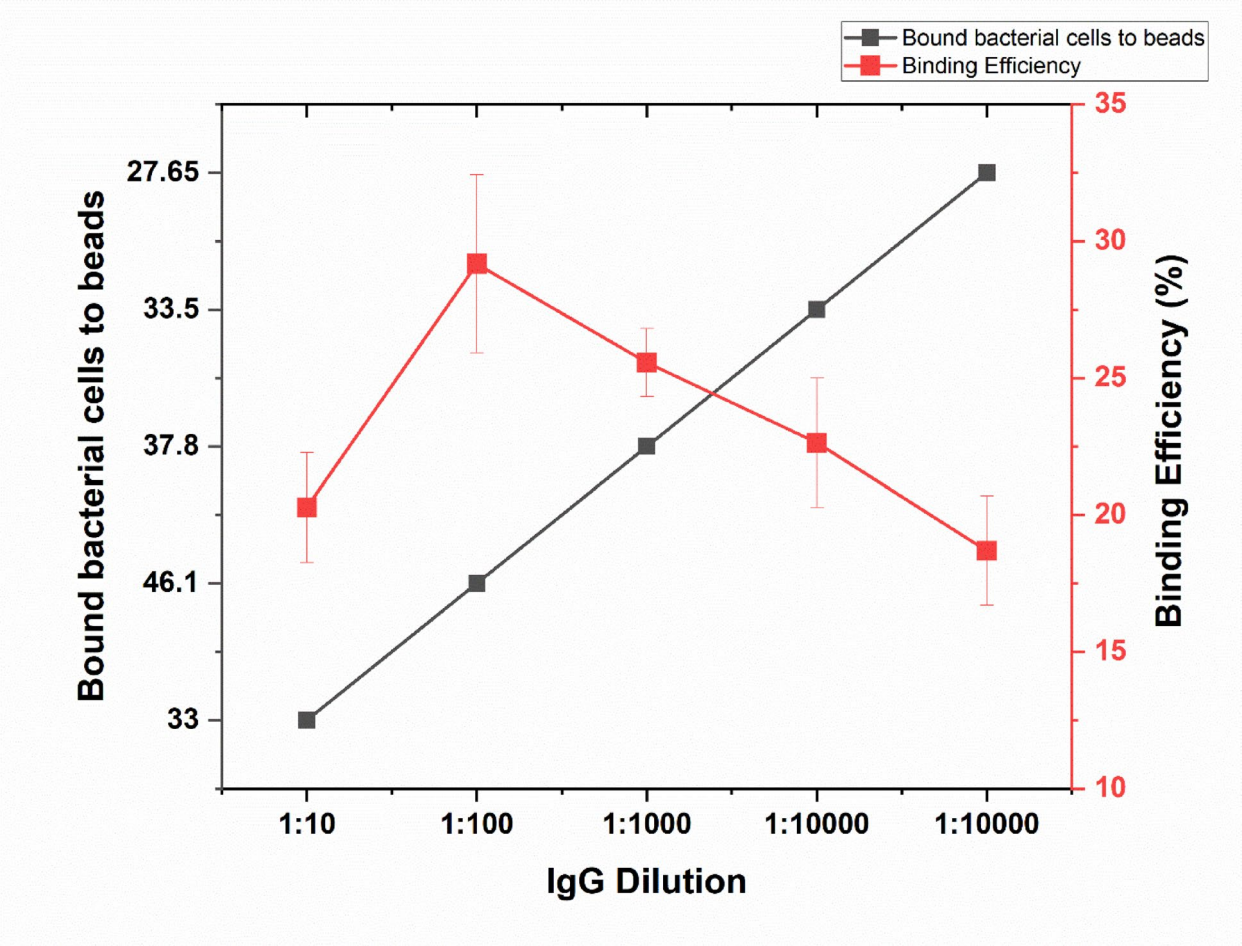
Effect of IgG Concentration on Bacterial Cell Binding to Magnetic Beads. The figure illustrates the influence of IgG dilution on the number of bacterial cells bound to magnetic beads (black line) and the corresponding binding efficiency (red line). Error bars represent the standard deviation of three independent replicates

With a 1:10 IgG dilution, the average number of bound bacteria was 33, corresponding to a binding percentage of 20.27%. At a 1:100 dilution, the average number of bound bacteria increased to 46.1, with a binding percentage of 29.17%. The 1:1000 dilution resulted in an average of 37.8 bound bacteria and a binding percentage of 25.56%. With a 1:10,000 dilution, the average number of bound bacteria was 33.5, yielding a binding percentage of 22.63%. Finally, at a 1:100,000 dilution, the average number of bound bacteria was 27.65, corresponding to a binding percentage of 18.69%, as shown in (Fig. 1 and supplementary Table 1). Each dilution was tested duplicate, indicating that binding efficiency of the immunomagnetic beads decreases with higher IgG dilution.

#### Influence of incubation temperature on binding of beads to rabbit IgG

At the initial measurement point (No added IgG), the average number of bound bacteria was 203, with no binding measurements taken. The control samples, which did not include beads, exhibited an average of 291 bound bacteria without any binding measurements.

**Fig. 2:**
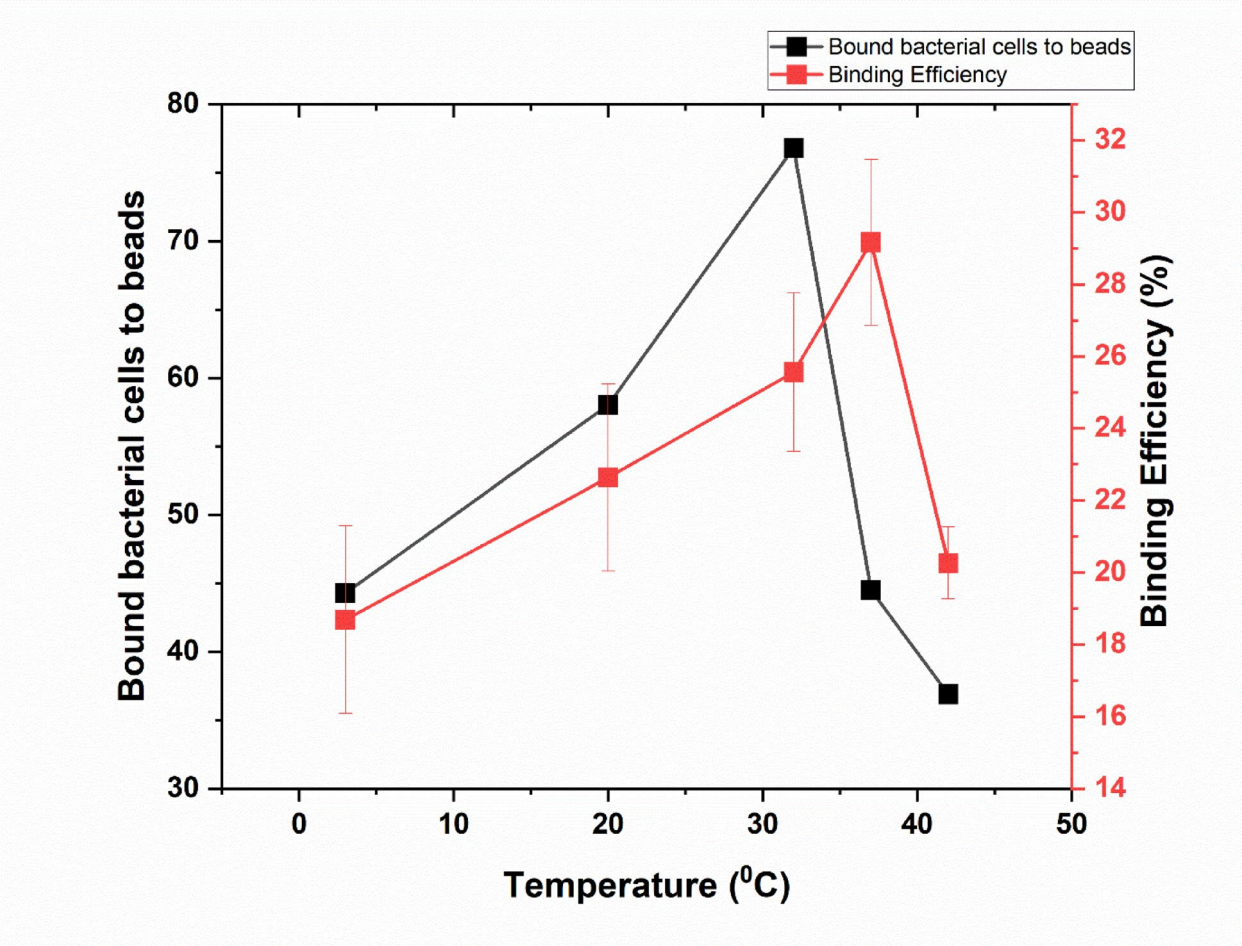
Influence of incubation temperature for 20 min on the binding of beads to rabbit IgG. The figure illustrates the influence of incubation temperature for 20 min on the number of bacterial cells bound to magnetic beads (black line) and the corresponding binding efficiency (red line). Error bars represent the standard deviation of three independent replicates

When the temperature was raised to 42 °C, the average number of bound bacteria was 36.9, with a binding percentage of 16.96%. However, as the temperature decreased to 37 °C and then to 32 °C, the average number of bound bacteria increased to 44.5 and 76.83, respectively, with binding percentages reaching 20.46% and 35.05%. At 20 °C, the average number of bound bacteria dropped to 58, with a binding percentage of 26.56%. At 3 °C, the average number of bound bacteria decreased further to 44.3, accompanied by a binding percentage of 20.28% as shown in (Fig. 2, and supplementary Table 2). In summary, the number of bound cells and the binding percentage increased progressively as the temperature decreased from 42 to 32 °C, where optimal cell binding was achieved. Beyond this point, both parameters began to decline with further decreases in temperature.

#### Influence of incubation temperature of binding cells to coated beads for 30 min

**Fig. 3:**
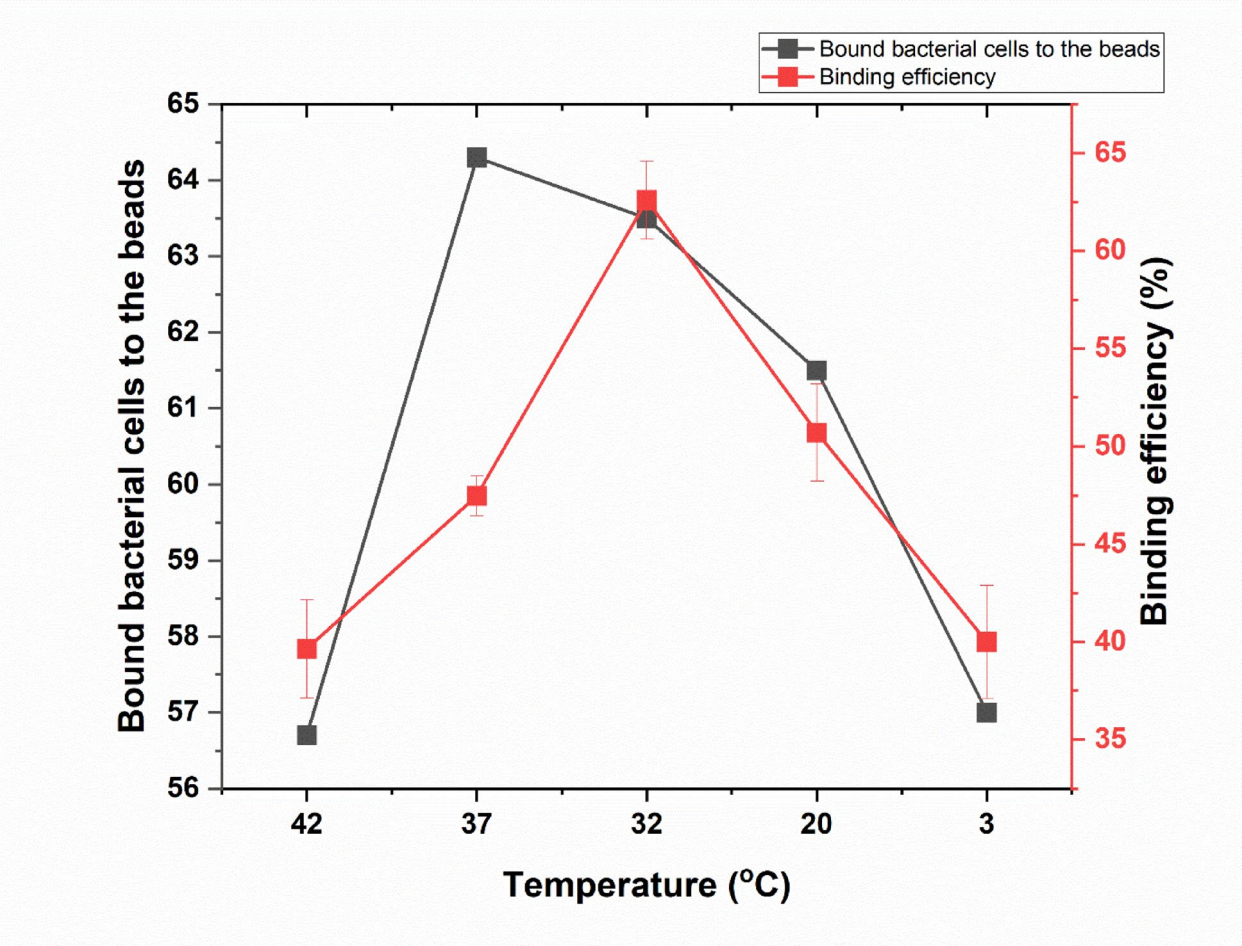
Influence of incubation temperature for 30 min on the binding of beads to rabbit IgG. The figure illustrates the influence of incubation temperature for 30 min on the number of bacterial cells bound to magnetic beads (black line) and the corresponding binding efficiency (red line). Error bars represent the standard deviation of three independent replicates

At the initial measurement point with no added IgG, the average number of bound bacteria was 94, with no binding measurements taken. When the temperature was raised to 42 °C, the average number of bound bacteria was 56.8, with a binding percentage of 39.65%. However, as the temperature decreased to 37 °C and then to 32 °C, the average number of bound bacteria increased to 64.3 and 63.5, respectively, with binding percentages reaching 47.47% and 62.6%. Conversely, further reductions in temperature led to a decline particularly in binding percentage. At 20 °C, the average number of bound bacteria was 61.5 with a binding percentage dropped to 50.7%. At 3 °C, the average number of bound bacteria decreased further to 57.1 accompanied by a binding percentage of 40.0% as shown in (Fig. 3 and supplementary Table 3). In conclusion, the number of bound bacteria and the binding percentage increased progressively as the temperature decreased from 42 to 32 °C, where optimal cell binding was achieved. Beyond this point, both parameters, especially the binding percentage, began to decline with further decreases in temperature as shown in (Table 3).

#### Evaluation of the purity of the magnetic beads

The binding efficiency of cells to beads was assessed under various conditions, including zero-time, control groups, and experimental groups. The percentage of bound bacteria was measured at multiple time points, and the average values were calculated along with the standard error of the mean (SEM) where applicable.

**Fig. 4:**
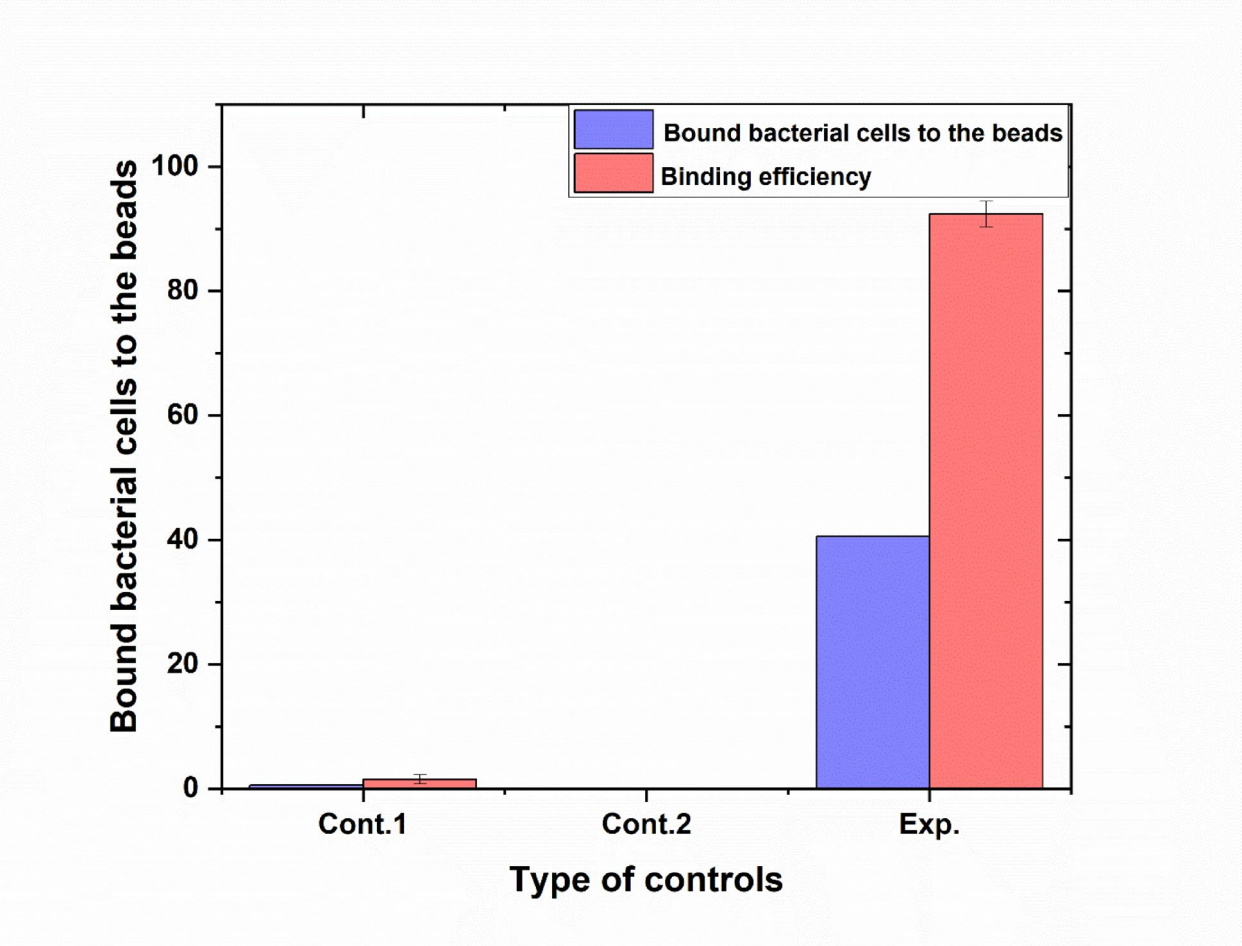
Evaluation of binding efficiency of *E. coli* cells to beads^.^ Control 1: represents a negative control, it contained coated beads with IgG but *E.coli* cells have not added to it,Control 2 also represent a negative control, but this one contained uncoated beads. *E.coli,* Exp represents the vial that contained the experimental IgG-coated beads. Error bars represent the standard deviation of three independent replicates

At zero time (baseline), the average percentage of bound bacteria was 43%. In the control group, the binding percentage was slightly higher, with an average of 44%. In Control 1, the binding was negligible, with an average of 1.0% (SEM = 0.76). Additional replicates showed minimal variation, with values ranging from 0.33 to 2.27%. Control 2 showed no binding, as expected, with all measurements consistently reporting 0% binding. In the experimental group, there was a significant increase in binding. The average binding percentage in the initial replicates was 43.33%, which was comparable to the zero time and control values. However, subsequent replicates showed a sharp increase, with values averaging 92.42% (SEM = 6.05), and individual measurements reaching as high as 98.48% and 86.36%. These results indicate that the experimental condition led to a marked increase in cell binding efficiency compared to both control groups and the baseline (zero time) as shown in as shown in (Fig. 4 supplementary Table 4).

#### Effect of pH value of 5% BSA in PBS on the binding of cells to the beads

**Fig. 5:**
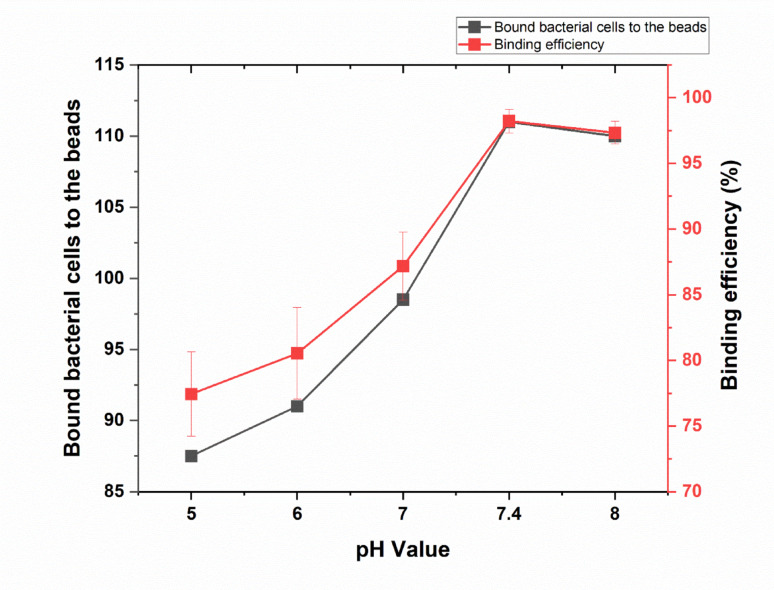
Influence of pH value of 5% BSA in PBS on the binding of beads to rabbit IgG. The figure illustrates the influence of pH value of 5% BSA in PBS on the number of bacterial cells bound to magnetic beads (black line) and the corresponding binding efficiency (red line). Error bars represent the standard deviation of three independent replicates

At the initial measurement point with no added IgG, the average number of bound bacteria was 113, with no binding measurements taken. The control samples, which did not include beads, exhibited the same average of 113 bound bacteria without any binding measurements. At the initial pH of 5, the average number of bound bacteria was 87.5, with a binding percentage of 77.43%. The average number of bound bacteria and binding percentage started to progressively increase with the increase of pH. At pH of 6, binding cells were 91 with binding percentage of 80.53%. At pH of 7, bound bacteria were 98.5 with binding percentage of 87.17%. At pH of 7.4, bound bacteria were 111 with binding percentage of 98.23%. However, at pH of 8, bound bacteria were dropped a little bit to 110 with binding percentage of 97.34%. Overall, the number of bound bacteria and the binding percentage increased exponentially with increasing in pH from 5 to 7.4, with a slight decline in these parameters at pH of 8. pH of 7.4 achieved the optimal number of bound bacteria and binding percentage (111 cells with 98.23% binding) as shown in (Fig. 5 and supplementary Table 5).

#### Effect of incubation time on the coating of rabbit anti E.coli O157 to the bead

**Fig. 6:**
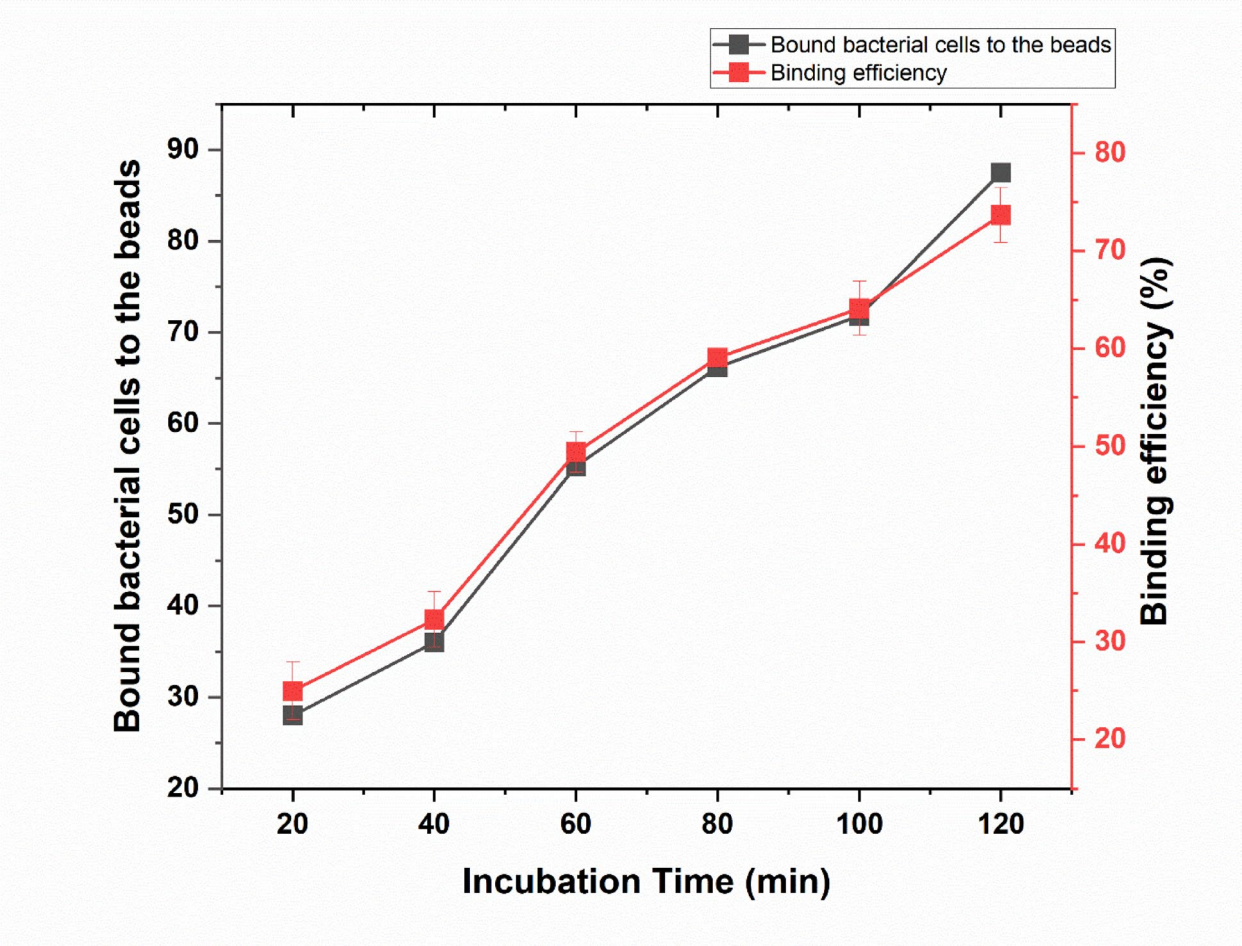
Influence of incubation time on the coating of rabbit anti *E.coli* O157 to the beads. The figure illustrates the influence of incubation time on the coating of rabbit anti *E.coli* O157 to the magnetic beads (black line) and the corresponding binding efficiency (red line). Error bars represent the standard deviation of three independent replicates

At the initial measurement point with no added IgG, the average number of bound bacteria was 103, with no binding measurements taken. The control samples, which did not include beads, exhibited the same average of 112.33 bound bacteria without any binding measurements. At the initial incubation time of 20 min, the average number of bound bacteria was 28, with a binding percentage of 25%. The average number of bound bacteria and binding percentage started to progressively increase with the increase of the incubation time. The number of bound bacteria increased to 36.17, 55.34, 66.17, 71.83, 87.5 with an increase in the incubation time to 40, 60, 80, 100, 120 min, respectively. Binding percentages increased from 32.29% after 40 min of incubation, to 49.40%, 59.07%, 64.13%, and 73.66% after incubation of 60, 80, 100, 120 min, respectively. To sum up, the number of bound bacteria and the binding percentage increased exponentially with increasing in the incubation time from 20 to 120 min. In which after 120 min, the optimal number of bound bacteria and binding percentages were achieved (87.5 cells with binding of 73.66%) as shown in (Fig. 6 and supplementary Table 6).

#### Maximum binding of captured E.coli from ground beef to the coated beads

**Fig. 7:**
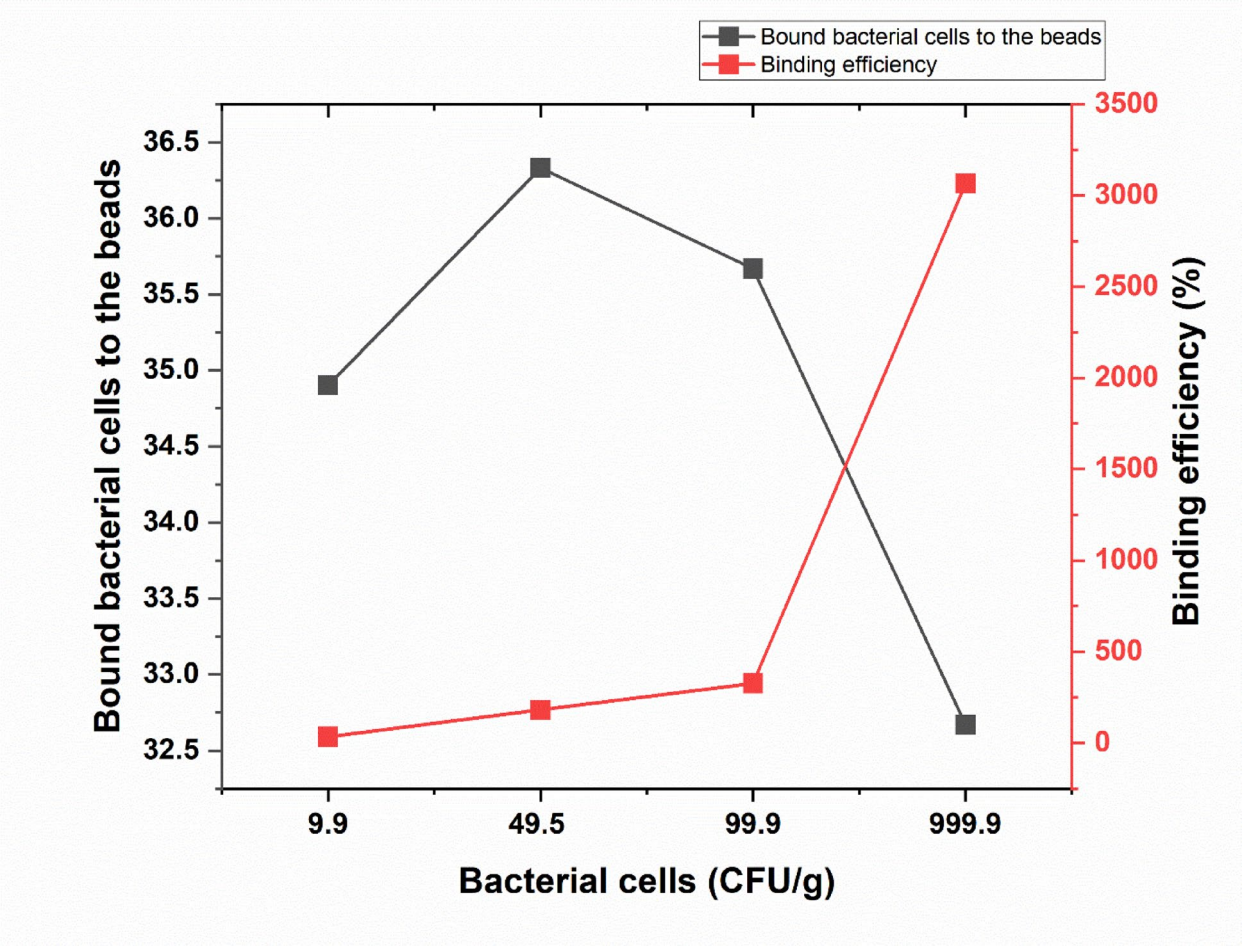
Influence of total bound bacteria (CFU/g) using coated beads to extract different concentration of *E.coli* O157:H7 cells from ground beef. The figure illustrates the influence of total bound bacteria (CFU/g) using coated beads to extract different concentration of *E.coli* O157:H7 to the magnetic beads (black line) and the corresponding binding efficiency (red line). Error bars represent the standard deviation of three independent replicates

The effect of varying concentrations of *Escherichia coli* O157 in ground beef on the binding efficiency to coated beads was evaluated. Control samples, which did not contain beads, consistently showed an average of 99 cells with no binding measurements. As the concentration of *E. coli* increased, a significant rise in bound cell counts was observed. At 9.9 CFU/g, the number of bound bacteria was 34.66, which increased exponentially with higher concentrations: at 49.5 CFU/g, the bound bacteria reached 181.65, and at 99.9 CFU/g, this rose further to 326.7. At the highest concentration of 999.9 CFU/g, the bound bacteria peaked at 3066.83 as shown in (Fig. 7 and supplementary Table 7).

Homogenates of unseeded frozen ground beef were tested on Violet Red Bile Agar (VRBA) with no *E. coli* colonies observed. Filtrates from these homogenates, further enriched and tested using latex agglutination, ELISA, and PCR, also showed no evidence of *E. coli* O157, confirming that the ground beef samples were free from viable or non-viable *E. coli* O157. This validates the efficiency of the freeze–thaw process used to eliminate *E. coli* O157 from the samples, while also demonstrating that increasing *E. coli* concentrations in ground beef correlates with enhanced binding to the coated beads.

## Discussion

In this study, we investigated the parameters influencing the binding efficiency of immunomagnetic beads in capturing target cells. Optimum binding condition is Immunomagnetic separation is a widely used technique for the isolation and enrichment of specific cell populations, relying on the selective binding of antibody-coated magnetic beads to surface antigens. The effectiveness of this approach is largely dependent on the density and functionality of the antibodies conjugated to the bead surface. By examining the impact of varying IgG concentrations, we aimed to optimize binding conditions and gain insights into the factors that enhance or hinder bead-cell interactions. The findings provide valuable guidance for improving the reliability and sensitivity of immunomagnetic separation protocols.

The binding efficiency of the immunomagnetic beads was observed to decrease with increasing IgG dilution, indicating that lower antibody concentrations on the bead surface result in reduced cell capture. This trend can be attributed to the decreased availability of functional binding sites at higher dilutions, which diminishes the likelihood of effective interactions between the beads and target cells. Conversely, higher concentrations of IgG (i.e., lower dilutions) enhance binding efficiency by increasing the density of antibody molecules on the bead surface, thereby promoting more robust and specific cell-bead interactions. These findings underscore the importance of optimizing IgG concentration to maximize the performance of immunomagnetic separation techniques. It was demonstrated that higher antibody densities on the beads lead to more robust and specific interactions (Wang et al. 1992). This phenomenon aligns with previous studies showing that the efficiency of IMS is highly dependent on the antibody density and binding conditions. For example, Chapman et al. (1994) reported that IMS could recover *E. coli* O157 from samples with as few as 2 CFU/g, highlighting the method’s sensitivity when optimal binding conditions are met (Chapman et al. 1994). Moreover, the use of beads coated with specific antibodies has been shown to significantly improve capture rates, especially when combined with optimized incubation and washing protocols (Wang et al. 1992).

Our results also indicate that incubation temperature significantly influences the binding efficiency of immunomagnetic beads to rabbit IgG. The highest binding efficiency was observed at 32 °C, with an average of 81.67 bound bacteria and a binding percentage of 35.05%. This optimal temperature likely facilitates the most favorable conditions for bead-cell interactions, possibly by maintaining the structural integrity and functional activity of the IgG molecules. Temperatures above or below 32 °C resulted in diminished binding efficiency, with minimal binding observed at 20 °C and 3 °C. These findings suggest that deviations from the optimal temperature impair the binding process, potentially due to denaturation of IgG at higher temperatures or reduced molecular mobility at lower temperatures. We clarified in that this relatively lower efficiency is attributed to partial denaturation of IgG or altered epitope–antibody interactions near physiological temperatures. This observation is consistent with existing literature that emphasizes the importance of maintaining optimal incubation conditions to ensure effective immunomagnetic separation. Our results are in agreement with reports of another research group (Tsai et al. 2015). Similarly, Wang et al. (1992) reported that maintaining appropriate physical conditions, including temperature, was essential for maximizing the recovery of *E. coli* O157 using immunomagnetic beads.

Evaluation of bead purity further confirmed the robustness of the optimized conditions. Baseline (zero time) and general control samples exhibited moderate binding percentages around 43–44%, indicating standard assay performance. In contrast, the negative controls (Control 1 and Control 2) confirmed the specificity of the method, showing negligible or zero binding. Most notably, the experimental group displayed a dramatic increase in binding efficiency, with some replicates reaching up to 98.48% and an average of 92.42%. This marked enhancement highlights the effectiveness of the optimized experimental conditions and validates the purity and functionality of the magnetic beads. Taken together, these findings emphasize the importance of precise controlling experimental parameters—particularly IgG concentration and incubation temperature—to maximize the efficiency and specificity of immunomagnetic separation (Safarik and Safarikova 2004; Frenea-Robin and Marchalot 2022; Desai 2018). Cheng et al. (2022) found that the capture efficiency of exosomes using immunomagnetic beads was significantly higher at neutral pH compared to acidic or alkaline conditions (Cheng et al. 2022). This suggests that neutral pH conditions enhance the binding interactions by maintaining the structural integrity of the antibodies and target molecules. In a study by Zhang et al. (2023), the binding efficiency of immunomagnetic beads to various cell types was optimized at pH 7.4. The researchers noted a decline in binding efficiency at both lower and higher pH levels, reinforcing the need for precise pH control in immunomagnetic separation protocols. We found that performing the blocking step at pH 8.0 improved the functional stability of the IgG conjugates on the bead surface. This alkaline environment maintains the native conformation and net negative charge of the antibody, reducing potential denaturation or aggregation while enhancing antigen-binding orientation. This is consistent with the findings of Sapsford et al. (2013), who reported that antibody binding efficiency to solid supports is enhanced at near-neutral to mildly basic conditions due to improved charge stabilization and hydrophobic interactions.

Regarding the blocking agent concentration, we selected 5% (w/v) BSA based on optimization experiments where 1%, 3%, and 5% concentrations were compared. Lower concentrations (1–3%) resulted in incomplete surface coverage and higher nonspecific bacterial adsorption, while 5% BSA produced the most consistent reduction in background binding without impairing specific antigen recognition. This is in agreement with the findings of Li et al. (2018), Talanta), who demonstrated that higher BSA concentrations (≥ 5%) significantly improve assay specificity by saturating unreacted sites on magnetic or polymeric bead surfaces.

Our results demonstrate a clear correlation between incubation time and the efficiency of antibody coating on the magnetic beads. Shorter incubation periods, such as 20 and 40 min, resulted in low binding percentages, likely due to incomplete adsorption of the rabbit anti-*E. coli* O157 antibodies onto the bead surface (Pedersen et al. 2013; Schlosser et al. 2007). As the incubation time increased, both the number of bound bacteria and the binding efficiency improved significantly, reaching optimal values at 120 min (Pedersen et al. 2013; Schlosser et al. 2007). This suggests that longer incubation periods provide sufficient time for antibodies to uniformly bind and stabilize on the bead surface, thereby enhancing their ability to capture target cells (Pedersen et al. 2013; Schlosser et al. 2007). This outcome indicates that binding efficiency is strongly influenced by the structural integrity of IgG and its proper orientation on the bead surface (Pedersen et al. 2013; Schlosser et al. 2007). These findings highlight the importance of optimizing incubation time during bead preparation to ensure effective antibody conjugation and maximum cell-binding efficiency (Pedersen et al. 2013; Schlosser et al. 2007). Zhang et al. (2023) reported that the kinetics of antibody adsorption and orientation are crucial for achieving high binding efficiency. Their study indicated that longer incubation periods favor a more complete and functionally accessible coating of antibodies on magnetic beads. In addition to surface density and orientation, the binding affinity of antibodies toward *E. coli* O157:H7 antigens plays a decisive role in determining IMS performance. High-affinity interactions (typically in the nanomolar range) promote rapid and stable antigen–antibody complex formation, which enhances bacterial capture even under low target concentrations (Wang et al*.*, 2017; Hyeon et al. 2018). Conversely, antibodies with suboptimal affinity may exhibit lower capture rates or higher dissociation during washing steps, leading to reduced recovery efficiency. The rabbit polyclonal IgG used in this study exhibited strong antigen-binding capacity, consistent with previously reported affinities of 1–10 nM for anti-O157 immunoglobulins (Zhao et al*.*, 2020). Thus, both antibody density and binding strength synergistically determine the effective capture and retention of *E. coli* O157:H7 cells during the IMS process.

Although the present study primarily focused on optimizing the coating and binding efficiency of IgG-coated magnetic beads, the quantitative detection capability of the IMS method was evaluated by comparison with published data. Previous studies have reported comparable detection limits for *E. coli* O157:H7 ranging between 15 and 20 CFU/g using optimized IMS or IMS–PCR approaches (Hyeon et al. 2018; Ma et al. 2023). The capture efficiency observed in this study suggests a similar sensitivity range, indicating that the optimized IMS process provides reliable bacterial recovery suitable for downstream detection and enumeration. Future work will include establishing a full calibration curve under standardized conditions to confirm the assay’s quantitative parameters.

The data clearly demonstrate a concentration-dependent relationship between the number of *E. coli* O157 cells present in ground beef and their binding efficiency to antibody-coated magnetic beads. As the bacterial load increased from 9.9 to 999.9 CFU/g, the number of bound bacteria rose exponentially, highlighting the sensitivity and effectiveness of the immunomagnetic separation method in capturing target cells even at low concentrations (Jayamohan et al. 2015; Nou et al. 2006). This strong correlation suggests that the coated beads are highly efficient in detecting *E. coli* O157 in complex food matrices such as ground beef, making this technique valuable for microbial food safety applications (Jayamohan et al. 2015; Nou et al. 2006). The observed relationship between bacterial concentration and bound bacteria closely follows the exponential capture kinetics reported by Yu et al. (1995) and López-Campos et al. (2012), both of which demonstrated similar nonlinear increases in binding efficiency with higher bacterial loads.

In the present study, the main focus was to optimize the immunomagnetic separation (IMS) process parameters including antibody coating, incubation time, and washing efficiency rather than to quantitatively validate detection limits under standardized analytical conditions. Therefore, LOD and LOQ values were not experimentally determined during this phase of the work, and it will be our future work. However, our optimized IMS conditions (1 mg IgG-coated magnetic beads per 1 mL sample, 20 min incubation at 32 °C) closely resemble those reported in prior IMS-based *E. coli O157:H7* detection studies. Published data indicate that similar systems achieve LOD values ranging between 10 and 30 CFU/mL and LOQ values between 25 and 50 CFU/mL under comparable experimental parameters. For instance, Yu et al. (1995) reported a detection limit of approximately 20 CFU/mL using Protein A–based magnetic beads, and Cudjoe and Krona (1997) observed an LOD of 15 CFU/mL when employing a similar antibody-bead conjugation approach. Finally, our findings confirm that the detection sensitivity of the bead-based assay scales with bacterial load, reinforcing its utility in food safety testing.

## Conclusion

Our study highlights the critical parameters of IgG concentration, incubation temperature, and incubation time in optimizing the binding efficiency of immunomagnetic beads. The concentration-dependent relationship between bacterial load and binding efficiency underscores the sensitivity and effectiveness of this method for detecting *E. coli* O157 in complex food matrices. These findings provide valuable insights for improving the performance of immunomagnetic separation techniques in various biomedical and food safety applications. Future research should explore the mechanistic underpinnings of these observations and extending these studies to different types of antibodies and target cells to enhance the applicability and robustness of immunomagnetic separation methods.

## Supplementary Information

Below is the link to the electronic supplementary material.

Supplementary Material 1

## Data Availability

No datasets were generated or analysed during the current study.

## References

[CR1] Ahmed AM, Shim YS, Moon DC, Kim SJ, Kang HY, Park YH (2020) Biofilm formation and antibiotic resistance patterns of *Escherichia coli* isolated from food samples. Food Control 112:107106

[CR2] Ashkenazi S, Cleary TG (1989) Rapid method to detect Shiga toxin and Shiga-like toxin I based on binding to globotriosyl ceramide (Gb3), their natural receptor. J Clin Microbiol 27:1145–1150. 10.1128/jcm.27.6.1145-1150.1989 2666433 10.1128/jcm.27.6.1145-1150.1989 PMC267516

[CR3] Bettelheim KA (2003) The non-O157 Shiga-toxigenic (verocytotoxigenic) *Escherichia coli*: under-rated pathogens. Crit Rev Microbiol 29:283–298. 10.1080/10408410390251050 10.1080/10408410601172172 17453930

[CR5] Chapman PA, Siddons CA, Cerdan Malo AT, Harkin MA (1994) A 1-year study of *Escherichia coli* O157:H7 in cattle, sheep, pigs and poultry. Epidemiol Infect 113:219–225 10.1017/s0950268897007826 PMC2808847 9363024

[CR6] Chen Q, Lin Z, Lin J, Ye J, Liu Y (2021) A dual-recognition immunomagnetic separation and colorimetric assay for sensitive detection of *Escherichia coli* O157:H7 using antibody-modified magnetic nanoparticles. Talanta 122291:1–8. 10.1016/j.talanta.2021.122291

[CR7] Cheng J, Zhu N, Zhang Y, Yu Y, Kang K, Yi Q, Wu Y (2022) Hedgehog-inspired immunomagnetic beads for high-efficient capture and release of exosomes. J Mater Chem B 10:4059–4069. 10.1039/d2tb00226d 35521754 10.1039/d2tb00226d

[CR8] Cudjoe KS, Krona R (1997) Detection of Salmonella in foods by immunomagnetic separation. J Microbiol Methods 30:143–151

[CR9] Desai JP (2018) The effect of magnetic bead size on the isolation efficiency of lung cancer cells in a serpentine microchannel. Biomed Microdev 26:7. 10.1007/s10544-023-00689-5 10.1007/s10544-023-00689-5 38175269

[CR10] Feng P (1995) *Escherichia coli* serotype O157:H7: novel vehicles of infection and emergence of phenotypic variants. Emerg Infect Dis 1:47–52. 10.3201/eid0102.950201 8903158 10.3201/eid0102.950202 PMC2626836

[CR11] Frenea-Robin M, Marchalot J (2022) Basic principles and recent advances in magnetic cell separation. Magnetochemistry 8:11. 10.3390/magnetochemistry8010011

[CR12] Harlow E, Lane D (1988a) *Antibodies: a laboratory manual*. Cold Spring Harbor Laboratory Press, Cold Spring Harbor, NY. https://archive.org/details/antibodieslabora0000unse

[CR13] Hyeon JY, Chon JW, Hwang IG, Kwak HS, Seo KH (2018) Improvement of detection of *Listeria monocytogenes* in food using selective enrichment and centrifugation at 16,000×g. Front Microbiol 9:136029988535

[CR14] Jayamohan H, Gale BK, Minson BJ, Lambert CJ, Gordon N, Sant HJ (2015) Highly sensitive bacteria quantification using immunomagnetic separation and electrochemical detection of guanine-labeled secondary beads. Sensors 15:12034–12052. 10.3390/s150512034 26007743 10.3390/s150512034 PMC4481928

[CR15] Li X, Li P, Lei J, Zhang Q, Zhang W (2018) Immunomagnetic separation combined with chemiluminescence for detection of *Escherichia coli* O157:H7 in foods. Talanta 178:854–86129136906

[CR16] Lim JY, Yoon JW, Hovde CJ (2010) A brief overview of *Escherichia coli* O157:H7 and its plasmid O157. J Microbiol Biotechnol 20:5–14. 10.4014/jmb.0907.07007 20134227 PMC3645889

[CR17] Liu Y, Chen W, Wang J (2014) Optimization of immunoassays for detection of foodborne pathogens: effects of temperature, antibody concentration, incubation time, and pH. Anal Biochem 452:29–35

[CR18] Liu Y, Wang J, Li S, Zhou Y (2023) Multifunctional magnetic nanocomposites for efficient immunomagnetic separation and detection of foodborne bacteria. Microchem J 108479:1–9. 10.1016/j.microc.2023.108479

[CR19] López-Campos G, Martínez-Suárez JV, Aguado-Urda M, López-Alonso V (2012) Detection, identification, and analysis of foodborne pathogens. Microarrays 1(3):1–24

[CR20] Lowry OH, Rosebrough NJ, Farr AL, Randall RJ (1951) Protein measurement with the Folin phenol reagent. J Biol Chem 193:265–275 14907713

[CR21] Ma Y, Wang X, Zhang C et al (2023) Improved magnetic bead-based immunoassay for sensitive detection of *Escherichia coli* O157:H7 in complex food matrices. Microchem J 188:108479. 10.1016/j.microc.2023.108479

[CR22] March SB, Ratnam S (1986) Sorbitol-MacConkey medium for detection of *Escherichia coli* O157:H7 associated with hemorrhagic colitis. J Clin Microbiol 23:869–872 3519658 10.1128/jcm.23.5.869-872.1986 PMC268739

[CR23] Nada HG, El-Tahan AS, El-Didamony G, Askora A, Abdel-Glil MY (2023) Detection of multidrug-resistant Shiga toxin-producing *Escherichia coli* O157:H7 carrying *stx1* and *stx2* virulence genes from food and cattle faeces in Egypt. Int J Food Microbiol 397:110219 10.1186/s12866-023-02873-2 PMC10176883 37173663

[CR24] Nou X, Arthur TM, Bosilevac JM, Brichta-Harhay DM, Guerini MN, Kalchayanand N, Koohmaraie M (2006) Improvement of immunomagnetic separation for *Escherichia coli* O157:H7 detection by the PickPen magnetic particle separation device. J Food Prot 69:2870–2874 17186652 10.4315/0362-028x-69.12.2870

[CR25] Paton AW, Paton JC (1998) Detection and characterization of Shiga toxigenic *Escherichia coli* by using multiplex PCR assays for stx1, stx2, eaeA, enterohemorrhagic *E. coli* hlyA, rfbO111, and rfbO157. J Clin Microbiol 36:598–602 9466788 10.1128/jcm.36.2.598-602.1998 PMC104589

[CR26] Paton AW, Paton JC (2004) Structure of Shiga toxin type 2 (Stx2) from *Escherichia coli* O157:H7. J Biol Chem 279:27513 10.1074/jbc.M401939200 15075327

[CR27] Pedersen KW, Kierulf B, Oksvold MP, Li M, Vlassov AV, Roos N, Neurauter A (2013) Isolation and characterization of exosomes using magnetic beads. BioProbes 71:10–13

[CR28] Pennington H (2010) *Escherichia coli* O157. Lancet 376:1428–1435. 10.1016/S0140-6736(10)60963-4 20971366 10.1016/S0140-6736(10)60963-4

[CR29] Rangel JM, Sparling PH, Crowe C, Griffin PM, Swerdlow DL (2005) Epidemiology of *Escherichia coli* O157:H7 outbreaks, United States, 1982–2002. Emerg Infect Dis 11:603–609. 10.3201/eid1104.041063 15829201 10.3201/eid1104.040739 PMC3320345

[CR30] Safarik I, Safarikova M (2004) Magnetic techniques for the isolation and purification of proteins and peptides. BioMagn Res Technol 2:7. 10.1186/1477-044X-2-7 15566570 10.1186/1477-044X-2-7 PMC544596

[CR31] Sapsford KE, Francis J, Sun S, Kostov Y, Rasooly A (2013) Miniaturized 96-well microplate-based fluorescence immunoassay for toxin detection. Anal Bioanal Chem 405:2339–2348

[CR32] Sayedahmed AA (2006) Development of methodology for quantitative detection of *E. coli* O157:H7 in ground beef via immunoassays and the polymerase chain reaction. Doctoral dissertation, University of Massachusetts Amherst.

[CR33] Schlosser G, Káčer P, Kuzma M, Szilágyi Z, Sorrentino A, Manzo C, Pizzano R, Malorni L, Pocsfalvi G (2007) Coupling immunomagnetic separation on magnetic beads with MALDI-TOF MS for detection of staphylococcal enterotoxin B. Appl Environ Microbiol 73:5358–5365. 10.1128/AEM.01136-07 10.1128/AEM.01136-07 PMC2074950 17827336

[CR35] Thayer DW, Boyd G (1999) Effect of freezing and thawing on survival of *Escherichia coli* O157:H7 in food. J Food Prot 62(10):1124–1127

[CR36] Tsai YL, Wang HC, Lin YC, Chou CH, Cheng CM (2015) Rapid and sensitive detection of *Escherichia coli* O157:H7 using immunomagnetic separation and enzyme-based colorimetric assay. J Food Sci 80:M2542–M2548. 10.1111/1750-3841.13042

[CR37] Wang G, Zhao T, Doyle MP (1992) Detection and enumeration of *Escherichia coli* O157:H7 in food by immunomagnetic separation and PCR. Appl Environ Microbiol 58:283–286

[CR38] Wang L, Chen S, Xu Z, Zhang W (2018) Enhanced detection of *E. coli* O157:H7 in food samples using immunomagnetic separation combined with gold nanoparticle-based colorimetric assay. Talanta 189:566–573. 10.1016/j.talanta.2018.09.043

[CR39] Wang F, Yang Q, Qu Y, Meng J, Zhang Y (2021) Rapid detection of *Escherichia coli* O157:H7 in ground beef using colorimetric LAMP assay. Food Control 123:107722. 10.1016/j.foodcont.2020.107722

[CR40] Wilson IG (1997) Inhibition and facilitation of nucleic acid amplification. Appl Environ Microbiol 63:3741–3751 9327537 10.1128/aem.63.10.3741-3751.1997 PMC168683

[CR41] Wu VCH, Fung DYC, Lin CS (2014) Application of immunomagnetic separation and real-time PCR for rapid detection of *Escherichia coli* O157:H7 in ground beef. Int J Food Microbiol 174:52–59. 10.1016/j.ijfoodmicro.2014.03.022

[CR42] Yu LS-L, Reed SA, Beuchat LR (1995) Use of immunomagnetic separation combined with colony immunoblot for detection of *Escherichia coli* O157:H7 in foods. Appl Environ Microbiol 61:3268–3273 7574637 10.1128/aem.61.9.3268-3273.1995 PMC167607

[CR43] Zhang X, Li Y, Wang H (2017) Rapid detection of pathogenic *Escherichia coli* using nanomagnetic immunoseparation combined with loop-mediated isothermal amplification. J Zhejiang Univ-Sci B 18(5):397–407. 10.1631/jzus.B1600358

[CR44] Zhang Y, Kang F, Yang Y, Li J, Chen E, Hong T, Zhao L, Du M (2023) pH-regulated strategy and mechanism of antibody orientation on magnetic beads for improving capture performance. Foods 11:3599. 10.3390/foods11223599 10.3390/foods11223599 PMC9689862 36429188

[CR45] Zhao Y, Zeng D, Yan C, Chen W, Ren J, Jiang Y, Jiang L, Xue F, Ji D, Tang F, Zhou M, Dai J (2020) Rapid and accurate detection of Escherichia coli O157:H7 in beef using microfluidic wax-printed paper-based ELISA. Analyst 145:2928–2935 10.1039/D0AN00224K 10.1039/d0an00224k 32159201

