# Supplementary material for: Optimizing immunomagnetic separation for efficient E. coli O157:H7 recovery and detection
Source: AMB Express. 2025 Dec 20;16:7. doi: 10.1186/s13568-025-01991-6 (PMC12834865; doi:10.1186/s13568-025-01991-6)
Supplement: Supplementary file 1 — Supplementary Material 1 [file 13568_2025_1991_MOESM1_ESM.docx]

**Supplementary file:**

**Supplementary Table.1. Effect of IgG concentration on the binding of cells to beads a, b.**

| **IgG** | **Bound cells to the beads** | | | | **Binding%** | | |
| --- | --- | --- | --- | --- | --- | --- | --- |
| **Dilution** | **1** | **2** | **3** | **ave** | **4** | **Ave** | **SEM** |
|  |  |  |  |  |  |  |  |
| 1:10 | 34 | 30 | 20 | 33 | 18.92 | 20.27 | 5.4 |
|  | 37 | 38 | 41 |  | 25.67 |  |  |
|  |  |  |  |  |  |  |  |
| 1:100 | 46 | 33 | 45 | 46.1 | 27.93 | 29.17 | 3.26 |
|  | 40 | 62 | 51 |  | 34.46 |  |  |
|  |  |  |  |  |  |  |  |
| 1:1000 | - | 36 | 36 | 37.8 | 24.32 | 25.56 | 1.24 |
|  | 46 | 29 | 43 |  | 26.80 |  |  |
|  |  |  |  |  |  |  |  |
| 1:10000 | - | 41 | 34 | 33.5 | 25 | 22.63 | 2.36 |
|  | 26 | 35 | 29 |  | 20.27 |  |  |
|  |  |  |  |  |  |  |  |
| 1:100000 | 38 | 30 | 40 | 27.65 | 24.32 | 18.69 | 5.63 |
|  | 21 | 14 | 23 |  | 13.06 |  |  |

a 1, 2, 3, represent the experiments, each performed in duplicates followed by the numerical average, 4 indicates percent of cells bound to beads.

b Control vials contained no beads. Cont.1 just cells-cont.2 to check the vials.

At time zero, mean CFU counts were 140 for the glass vials and 148 for the beads (without IgG). Values were adjusted by subtracting baseline counts from experimental counts. Control experiments confirmed no effect of glass vials, beads, temperature, or IgG on CFU counts.

**Supplementary Table. 2. Influence of incubation temperature for 20 min on binding of beads to rabbit IgGa.**

| **Temp.(oC)** | **Bound cells to the beads** | | | | **Binding%** | | |
| --- | --- | --- | --- | --- | --- | --- | --- |
|  | **1** | **2** | **3** | **ave** | **4** | **Ave** | **SEM** |
| Zero time | 232 | 188 | 191 | 203 |  |  |  |
| cont. | 219 | 224 | 215 | 219 |  |  |  |
|  |  |  |  |  |  |  |  |
| 42 | 33 | 45 | 40 | 36.9 | 17.96 | 16.96 | 1.06 |
|  | 41 | 38 | 25 |  | 15.83 |  |  |
|  |  |  |  |  |  |  |  |
| 37 | 41 | 35 | 43 | 44.5 | 18.1 | 20.46 | 2.36 |
|  | 44 | 49 | 58 |  | 22.83 |  |  |
|  |  |  |  |  |  |  |  |
| 32 | 87 | 80 | 78 | 76.8 | 37.29 | 35.05 | 2.25 |
|  | 55 | 86 | 75 |  | 32.8 |  |  |
|  |  |  |  |  |  |  |  |
| 20 | 56 | 85 | 64 | 58 | 31.2 | 26.56 | 2.65 |
|  | 51 | 60 | 33 |  | 21.91 |  |  |
|  |  |  |  |  |  |  |  |
| 3 | 59 | 48 | 44 | 44.3 | 22.9 | 20.28 | 2.61 |
|  | 36 | 34 | 46 |  | 17.66 |  |  |
|  |  |  |  |  |  |  |  |

a 1, 2, 3, represent the tropic experiments, each performed in duplicates followed by the numerical average, 4 indicates percent of cells bound to beads.

b Control vials contained no beads.

**Supplementary Table. 3. Influence of incubation temperature of binding cells to coated beads for 30 mina.**

| **Temp.(oC)** | **Bound cells to the beads** | | | | **Binding%** | | |  |  |  |  |  |
| --- | --- | --- | --- | --- | --- | --- | --- | --- | --- | --- | --- | --- |
|  | **1** | **2** | **3** | **Ave** | **4** | **Ave** | **SEM** |  |  |  |  |  |
| Zero time | 90 | 97 | 97 | 94 |  |  |  |  |  |  |  |  |
|  |  |  |  |  |  |  |  |  |  |  |  |  |
| 42- cont. | 96 | 95 | - | 56.7 |  |  |  |  |  |  |  |  |
|  | 40 | 35 | 25 |  | 35.1 | 39.65 | 2.55 |  |  |  |  |  |
|  | 45 | 35 | 46 |  | 44.21 |  |  |  |  |  |  |  |
|  |  |  |  |  |  |  |  |  |  |  |  |  |
| 37- Cont. | 91 | 108 | - | 64.3 |  |  |  |  |  |  |  |  |
|  | 48 | 50 | 61 |  | 53.53 | 47.47 | 1 |  |  |  |  |  |
|  | 44 | 43 | 36 |  | 41.41 |  |  |  |  |  |  |  |
|  |  |  |  |  |  |  |  |  |  |  |  |  |
| 32- Cont. | 83 | 82 | - | 63.5 |  |  |  |  |  |  |  |  |
|  | 44 | 57 | 68 |  | 68.7 | 62.6 | 2 |  |  |  |  |  |
|  | 45 | 55 | 39 |  | 56.5 |  |  |  |  |  |  |  |
|  |  |  |  |  |  |  |  |  |  |  |  |  |
| 20- Cont. | 95 | 95 |  | 61.5 |  |  |  |  |  |  |  |  |
|  | 43 | 57 | 61 |  | 56.49 | 50.7 | 2.5 |  |  |  |  |  |
|  | 41 | 45 | 42 |  | 44.91 |  |  |  |  |  |  |  |
|  |  |  |  |  |  |  |  |  |  |  |  |  |
| 3 - Cont. | 104 | 86 | - | 57 |  |  |  |  |  |  |  |  |
|  | 39 | 32 | 26 |  | 34.03 | 40.00 | 2.9 |  |  |  |  |  |
|  | 34 | 46 | 51 |  | 45.97 |  |  |  |  |  |  |  |

a 1, 2, 3, represent the tropic experiments, each performed in duplicates followed by the numerical average, 4 indicates percent of cells bound to beads.

b Control vials contained no beads. At time zero, mean CFU values were adjusted by subtracting baseline counts from experimental counts.

**Supplementary Table. 4. Evaluation of binding efficiency of *E. coli* cells to beadsa**.

| **Type of** | **Bound cells to beads** | | | | **%Binding** | | |  |  |  |  |  |
| --- | --- | --- | --- | --- | --- | --- | --- | --- | --- | --- | --- | --- |
| **controls** | **1** | **2** | **3** | **Ave** | **4** | **Ave** | **SEM** |  |  |  |  |  |
| Zero time | 46 | 44 | 40 | 43 |  |  |  |  |  |  |  |  |
|  |  |  |  |  |  |  |  |  |  |  |  |  |
| Cont. | 49 | 44 | 39 | 44 |  |  |  |  |  |  |  |  |
|  |  |  |  |  |  |  |  |  |  |  |  |  |
| Cont.1 | 0.0 | 1.0 | 2.0 | 0.6 | 2.27 | 1.51 | 0.76 |  |  |  |  |  |
|  | 1.0 | 0.0 | 0.0 |  | 0.75 |  |  |  |  |  |  |  |
|  |  |  |  |  |  |  |  |  |  |  |  |  |
| Cont.2 | 0.0 | 0.0 | 0.0 | 0.0 | 0.0 | 0.0 | 0.0 |  |  |  |  |  |
|  | 0.0 | 0.0 | 0.0 | 0.0 | 0.0 |  |  |  |  |  |  |  |
|  |  |  |  |  |  |  |  |  |  |  |  |  |
| Exp. | 43 | 48 | 39 | 40.6 | 98.48 | 92.42 | 2.05 |  |  |  |  |  |
|  | 39 | 42 | 33 |  | 86.36 |  |  |  |  |  |  |  |

**Control 1: represents a negative control, it contained coated beads with IgG bu*t E.col*i cells have not added to it.**

**Control 2 also represent a negative control, but this one contained uncoated beads*. E.coli* , Exp represents the vial that contained the experimental IgG-coated beads.**

a 1, 2, 3, represent the tropic experiments, each performed in duplicates followed by the numerical average, 4 indicates percent of cells bound to beads.b Control vials contained no beads. Cont.1: Assay for residual cells in the vial attached to the vial's wall. The vial contained 1 x 103 cells and the coated beads in 2.0 ml of 5% BSA in PBS. the vials were incubated at 32oC for 30 min. And then the beads were concentrated magnetically to the vial's wall. The supernatant was removed and then the beads were resuspended into 1.0 ml of PBS. Plate counts were obtained as described above. Cont.2: (Negative control) Assay for the effect of the beads without IgG on the binding of cells to the beads. Washed beads were incubated with 1 x 103 cells/ ml in a total volume of 2.0 ml of BSA in PBS at 32oC for 30 min. Beads were then concentrated using the previous method and then resuspended into 1.0 ml PBS. Plate counts were obtained as described above. At time zero, mean CFU values were adjusted by subtracting baseline counts from experimental counts.

**Supplementary Table. 5. Effect of pH value of 5% BSA in PBS on the binding of cells to the beadsa.**

| **pH Value** | **Bound cells to the beads** | | | | **Binding%** | | |  |  |  |  |  |
| --- | --- | --- | --- | --- | --- | --- | --- | --- | --- | --- | --- | --- |
|  | **1** | **2** | **3** | **Ave** | **4** | **ave** | **SEM** |  |  |  |  |  |
| Zero time | 119 | 107 | - | 113 |  |  |  |  |  |  |  |  |
| Cont. | 116 | 119 | 104 | 113 |  |  |  |  |  |  |  |  |
|  |  |  |  |  |  |  |  |  |  |  |  |  |
| 5.0 | 82 | 99 | 89 | 87.5 | 79.65 | 77.43 | 3.21 |  |  |  |  |  |
|  | 97 | 75 | 83 |  | 75.22 |  |  |  |  |  |  |  |
|  |  |  |  |  |  |  |  |  |  |  |  |  |
| 6.0 | 106 | 90 | 89 | 91 | 84.07 | 80.53 | 3.54 |  |  |  |  |  |
|  | 108 | 84 | 96 |  | 76.99 |  |  |  |  |  |  |  |
|  |  |  |  |  |  |  |  |  |  |  |  |  |
| 7.0 | 93 | 93 | 87 | 98.5 | 80.53 | 87.17 | 2.63 |  |  |  |  |  |
|  | 115 | 99 | 104 |  | 93.8 |  |  |  |  |  |  |  |
|  |  |  |  |  |  |  |  |  |  |  |  |  |
| 7.4 | 119 | 116 | 101 | 111 | 99.11 | 98.23 | 0.9 |  |  |  |  |  |
|  | 109 | 117 | 104 |  | 97.34 |  |  |  |  |  |  |  |
|  |  |  |  |  |  |  |  |  |  |  |  |  |
| 8.0 | 113 | 119 | 95 | 110 | 96.46 | 97.34 | 0.86 |  |  |  |  |  |
|  | 113 | 113 | 107 |  | 98.23 |  |  |  |  |  |  |  |

a 1, 2, 3, represent the tropic experiments, each performed in duplicates followed by the numerical average, 4 indicates percent of cells bound to beads.

b Control vials contained no beads. At time zero, mean CFU values were adjusted by subtracting baseline counts from experimental counts.

**Supplementary Table. 6. Effect of incubation time on the coating of rabbit anti *E.coli* O157 to the beadsa.**

| **Incubation Time (min)** | **Bound cells to the beads** | | | | **Binding%** | | |  |  |  |  |  |
| --- | --- | --- | --- | --- | --- | --- | --- | --- | --- | --- | --- | --- |
|  | **1** | **2** | **3** | **ave** | **4** | **ave** | **SEM** |  |  |  |  |  |
| Zero time | 105 | 109 | 95 | 103 |  |  |  |  |  |  |  |  |
| Cont. | 101 | 115 | 121 | 112.33 |  |  |  |  |  |  |  |  |
|  |  |  |  |  |  |  |  |  |  |  |  |  |
| 20 | 32 | 28 | 34 | 28 | 27.98 | 25.00 | 2.98 |  |  |  |  |  |
|  | 24 | 28 | 22 |  | 22.02 |  |  |  |  |  |  |  |
|  |  |  |  |  |  |  |  |  |  |  |  |  |
| 40 | 32 | 37 | 30 | 36 | 29.49 | 32.29 | 2.83 |  |  |  |  |  |
|  | 40 | 36 | 42 |  | 35.12 |  |  |  |  |  |  |  |
|  |  |  |  |  |  |  |  |  |  |  |  |  |
| 60 | 53 | 65 | 55 | 55.3 | 51.49 | 49.40 | 2.08 |  |  |  |  |  |
|  | 53 | 57 | 49 |  | 47.32 |  |  |  |  |  |  |  |
|  |  |  |  |  |  |  |  |  |  |  |  |  |
| 80 | 67 | 62 | 71 | 66.17 | 59.52 | 59.07 | 0.45 |  |  |  |  |  |
|  | 64 | 70 | 63 |  | 58.63 |  |  |  |  |  |  |  |
|  |  |  |  |  |  |  |  |  |  |  |  |  |
| 100 | 68 | 67 | 61 | 71.83 | 58.33 | 64.13 | 2.8 |  |  |  |  |  |
|  | 78 | 74 | 83 |  | 69.94 |  |  |  |  |  |  |  |
|  |  |  |  |  |  |  |  |  |  |  |  |  |
| 120 | 78 | 83 | 75 | 87.5 | 70.24 | 73.66 | 2.8 |  |  |  |  |  |
|  | 82 | 88 | 89 |  | 77.08 |  |  |  |  |  |  |  |

a 1, 2, 3, represent the tropic experiments, each performed in duplicates followed by the numerical average, 4 indicates percent of cells bound to beads.

b Control vials contained no beads. At time zero, mean CFU values were adjusted by subtracting baseline counts from experimental counts.

**Supplementary Table.7.** **Total bound bacteria using coated beads to extract different concentration of *E.coli* O157:H7 cells from ground beefa.**

| **CELLS** | **Plate counts of Bound cells** | | | | **Total count** | | |  |
| --- | --- | --- | --- | --- | --- | --- | --- | --- |
| **CFU/g** | **1** | **2** | **3** | **ave** | |  | **SEM** | |
| Cont. | 107 | 97 | 93 | 99 | |  |  | |
|  |  |  |  |  | |  |  | |
| 9.9 | 41 | 34 | 37 | 34.9 | | 34.66 | 2.66 | |
|  | 39 | 33 | 24 |  | |  |  | |
|  |  |  |  |  | |  |  | |
| 49.5 | 41 | 30 | 33 | 36.33 | | 181.65 | 2.32 | |
|  | 42 | 27 | 45 |  | |  |  | |
|  |  |  |  |  | |  |  | |
| 99.9 | 44 | 31 | 35 | 35.67 | | 326.7 | 4 | |
|  | 25 | 32 | 29 |  | |  |  | |
|  |  |  |  |  | |  |  | |
| 999.9 | 44 | 31 | 35 | 32.67 | | 3066.83 | 3 | |
|  | 30 | 43 | 31 |  | |  |  | |

a 1, 2, 3, represent the tropic experiments, each performed in duplicates followed by the numerical average, 4 indicates percent of cells bound to beads.

b Control vials contained no beads. At time zero, mean CFU values were adjusted by subtracting baseline counts from experimental counts.
